# Treatment decision-making process after an anterior cruciate ligament injury: patients’, orthopaedic surgeons’ and physiotherapists’ perspectives

**DOI:** 10.1186/s12891-022-05745-4

**Published:** 2022-08-16

**Authors:** Hanna Tigerstrand Grevnerts, Barbro Krevers, Joanna Kvist

**Affiliations:** 1 Unit of Physiotherapy, Department of Health, Medicine and Caring Sciences, Linköping University, Linkoping, Sweden; 2 Division of Physiotherapy, Department of Activity and Health, Linköping University, Linkoping, Sweden; 3 Department of Health, Medicine and Caring Sciences, Unit of Health Care Analysis and National Centre for Priorities in Health, Linköping University, Linkoping, Sweden; 4 Center for Medical Image Science and Visualization (CMIV), Department of Health, Medicine and Caring Sciences, Linköping University, Linkoping, Sweden; 5 Stockholm Sports Trauma Research Center, Dept of Molecular Medicine & Surgery, Karolinska Institute, Stockholm, Sweden

**Keywords:** Treatment decision, Shared decision making, ACL injury, ACL reconstruction

## Abstract

**Objective:**

To investigate the treatment decision-making process after an anterior cruciate ligament (ACL) injury from patients’, orthopaedic surgeons’ and physiotherapists’ perspectives.

**Methods:**

The study is a part of the NACOX study, which is designed to describe the natural corollaries after ACL injury. For the present study, a subgroup 101 patients were included. Patients, their orthopaedic surgeons and their physiotherapists, answered a Shared Decision-Making Process (SDMP) questionnaire, when treatment decision for ACL reconstruction surgery (ACLR) or non-reconstruction (non-ACLR) was taken. The SDMP questionnaire covers four topics: “ *informed patient*”, “ *to be heard*”, “ *involvement*” and “ *agreement*”.

**Results:**

Most (75–98%) patients considered their needs met in terms of *being heard* and *agreement* with the treatment decision. However, fewer in the non-ACLR group compared to the ACLR group reported satisfaction with *information* from the orthopaedic surgeon (67% and 79%), or for their own *involvement* in the treatment decision process (67% and 97%).

**Conclusion and practice implications:**

Most patients and caregivers considered that patients’ needs to be informed, heard and involved, and to agree with the decision about the treatment process, were fulfilled to a high extent. However, patients where a non-ACLR decision was taken experienced being involved in the treatment decision to a lower extent. This implies that the non-ACLR treatment decision process needs further clarification, especially from the patient involvement perspective.

**Supplementary Information:**

The online version contains supplementary material available at 10.1186/s12891-022-05745-4.

## Introduction

After an anterior cruciate ligament (ACL) injury, a decision for surgical reconstruction of the ACL (ACLR) or non-surgical (non-ACLR) treatment has to be taken. It is recommended that ACLR should be considered when the patient suffers from functional instability, has high activity demands and/or has a concomitant injury that should be treated with initial surgery [1–5]. It seems, from previous research, that high activity demands are the most important factor for the treatment decision [6–8], but to authors knowledge, neither the decision-making process nor the collaboration between patient and caregiver have been clearly described.

The decision about non-surgical or surgical treatment after ACL injury is complex, with low evidence for the best treatment [5, 9, 10]. For non-surgical treatment, rehabilitation lasts approximately four to five months, and the patient should probably be recommended not to return to contact and pivoting sports [11]. For athletes, regardless of ACLR or non-surgical treatment, there is an option to adjust sports participation and reduce the risk of long-term consequences related to the injury. If the non-surgically treated patient experiences functional limitations, there is also an option for delayed ACLR. On the other hand, an early ACLR can be performed when the patient aims to return to contact and pivoting sports [12]. The disadvantages of ACLR are the increased risk of knee problems and new injuries after returning to sports [13] but also the long rehabilitation time, usually consisting of a time of pre-rehabilitation [14] and at least nine months post ACLR [11]. Priorities for the young athlete, like a career in sports, education, family and social support etc. have to be considered. The patient’s preference, cost-effectiveness and risk assessment of the chosen treatment must also be taken into consideration [15]. In addition, the ability of the stakeholders to make the decision must be assessed, as well as the appropriateness of the decision itself [15, 16].

We have shown previously that orthopaedic surgeons and physiotherapists consider each other’s assessments to be important in the choice of treatment, and both professions also consider the patient’s wishes to be important [6]. Shared decision-making (SDM) enhances patients’ involvement in the treatment decision and results in a better treatment outcome compared to medicine’s traditional form of paternal decision-making [16]. SDM is most relevant in complex situations when: 1) there is more than one treatment option, 2) the scientific evidence for either option is low, 3) treatment options show a similar balance between benefits and harms and 4) possible benefits and harms affect patients differently [17].

In cases of complex treatment decisions with limited evidence for which treatment is superior, the importance of giving information about options, possible side effects and risks, and an exploration of the patient’s preferences, is enhanced [18]. There is a fine balance in informing the patient sufficiently, without overwhelming them with information. An SDM strategy in which healthcare personnel balance between sharing the information that the patient requests, in a clear and comprehensible way, and also managing the emotional responses that might come up, is recommended [18].

There are some studies of patients’ perspectives regarding choice of treatment after an ACL injury [7, 8, 19, 20], but to the authors´ knowledge, studies on a decision-making process that involves the perspectives of the patient, the orthopaedic surgeon and the physiotherapist are lacking. Hence, in order to provide evidence-based care and shared decision-making about the choice of treatment for patients with an ACL injury, we need to further investigate how the treatment decision-making process is experienced by patients, orthopaedic surgeons and physiotherapists. Therefore, this study aims to investigate the treatment decision-making process after an ACL injury from patients’, orthopaedic surgeons’ and physiotherapists’ perspectives, with a focus on information, communication, involvement and agreement.

## Methods

This explorative, observational questionnaire study is part of a prospective multicentre cohort study – the NACOX study – which is designed to describe the natural corollaries after ACL injury. One of the five predefined main objectives for the NACOX study was to describe the decision-making process for treatment and to evaluate patient satisfaction with the decision that was made [21].

The NACOX study is prospectively registered (NCT02931084) and ethical approval was granted by the Ethical Review Board in Linkoping, Dnr: 2016/44/31, 2018/123–32. All included healthcare personnel were included by informed consent, and patients gave informed and written consent.

### Participants and settings

Between June 2016 and October 2018, patients were recruited from six different orthopaedic clinics in cities of various sizes (urban and suburban environments) in Sweden. The study was conducted in a healthcare setting that is partially publicly financed and partially insurance financed. Inclusion criteria for the NACOX study were: ACL injury verified by MRI or clinical examination, sustained no more than six weeks prior to inclusion, and age between 15 and 40 years at the time of the ACL injury. Patients were excluded if they had a previous ACL injury to the same knee, fractures that required separate treatment, if they were unable to understand written and spoken Swedish, had cognitive impairments or other illnesses or injuries that impaired function (e.g. fibromyalgia, rheumatic diseases or other diagnoses associated with chronic pain).

Inclusion criteria for the current analysis were: 1) a treatment decision had been taken during the first 12 months after injury and 2) the Shared Decision-Making Process (SDMP) questionnaire (described below) had to be answered by all three parties, i.e. the patient, orthopaedic surgeon and physiotherapist.

In total, 275 patients (52% male, mean age 25 years at injury) with ACL injury were included in the NACOX study, and 156 of them (57%) underwent ACLR during the first year. This ratio of ACLR and non-ACLR treatment is in line with the national proportions in Swedish (country) healthcare [22].

### Questionnaires

The SDMP questionnaire is based on the CollaboRATE framework [23], and the study-specific SDMP was modified by the authors, in order to fit all three parties. The framework aims to shift the focus away from terms such as patient satisfaction and outcome, and instead focus on the process of treatment decision-making from a patient’s perspective [23]. The study-specific SDMP questionnaire was developed to address both patients and healthcare personnel, with modifications of the questions to fit all three parties.

The questions included in the SDMP questionnaire were grouped according to four main topics and labelled: “*informed patient”*, “*to be heard”,* “*involvement”* and *“agreement”.* The patient questionnaire included ten questions, and the orthopaedic surgeon and physiotherapist questionnaires included five questions each. The answers were on a five-point Likert scale indicating agreement: *“To a very high extent”, “To a high extent”, “Neither high nor low extent”, “To a low extent”, “To a very low extent”.* There was also an option to answer *“I do not know”* and some questions had an extra answer option, e.g. to indicate when the physiotherapist or orthopaedic surgeon was not involved in the process (Appendix 1).

The SDMP questionnaire was designed as an informative tool at a group level. The face validity of the questionnaires was tested for comprehensibility and possible redundancy, by an expert panel consisting of orthopaedic surgeons (*n* = 2) and physiotherapist (*n* = 2) who were active in treatment decision-making for patients with ACL injuries. One senior researcher, experienced in shared decision-making process research was also included. The orthopaedic surgeons (*n* = 2) had more than 20 years of experience in taking ACL treatment decisions. The physiotherapists had 2 and 33 years of experience, respectively, in the field of ACL injuries, research and treatments of this group of patients. Furthermore, all three authors are well experienced in instrument development.

The International Knee Documentation Committee’s Subjective Knee Evaluation Form (IKDC-SKF) is a knee-generic questionnaire with 18 items addressing symptoms, function and sports participation. The results provide a score from 0–100, where 100 equates to full function. The Swedish (country) version has been tested on patients with ACL injuries with good results [24] and the patient acceptable symptom state score has previously been defined as 75.9 points [25].

### Procedure

In the NACOX study, multiple questionnaires were sent to the patients. They were sent via text message weekly for the first six weeks, fortnightly from week seven to week 24 and monthly from month seven to month 12 after the initial injury [21]. Answers to these questionnaires indicated when a decision about surgical or non-surgical treatment had been made. At that time, a specific questionnaire about the shared decision-making process, i.e., the SDMP questionnaire, was sent to the patient, the treating orthopaedic surgeon, and the physiotherapist. Patients also answered a question about whether the chosen treatment matched their preferences, with answer options yes/no. All questionnaires were sent to the patient and the treating orthopaedic surgeon and physiotherapist, mainly using a secure web-based survey system (esMaker: version 3.0 © Entergate AB) using the short message system (SMS) or email. In some cases, the questionnaires were administered on paper.

At 12 months after injury for the patients who had non-surgically treated ACL, or 12 months after ACL reconstruction (ACLR) for the patients who had surgically treated ACL, the patients were sent a questionnaire including the IKDC-SKF [24, 26]. At the same follow-up time (12 months after injury or ACLR), patients answered a question about whether they would choose the same treatment for their knee injury if they had to choose again.

### Analysis

The answers to the SDMP questionnaire were analysed in three categories: 1) “To a high extent”, which included responses “to a very high extent” and “to a high extent”, 2) *“*Neither high nor low extent” and 3) “To a low extent”, which included responses “to a low extent” and “to a very low extent”. Extra response alternatives, e.g. “I do not know” and “was not involved in the decision” or “have not seen a physiotherapist/orthopaedic surgeon”, were excluded from the analyses, but are reported in Appendix 2.

An analysis of the representativeness of the study group was conducted by comparing the included patients with those who had been excluded due to all three parties not having answered the questionnaire or not having been involved in the decision-making process. The two groups were compared in terms of age, sex, and activity level before injury (Tegner Activity Scale score). Treatment group (non-ACLR or ACLR within one year after injury) was also compared between the groups. Group comparisons were analysed using the Chi-Square test, the Mann–Whitney U-test and the T-test, and a *p*-value of ≤ 0.05 was considered significant.

## Results

The study specific SDMP questionnaire was answered by 205 patients. For these 205 patients, 171 orthopaedic surgeons’ questionnaires (21 orthopaedic surgeons) and 167 physiotherapists’ questionnaires (60 physiotherapists) were answered. Since inclusion of patients was done at the orthopaedic clinic, all patients met the orthopaedic surgeons there, but were free to contact a physiotherapist elsewhere to begin rehabilitation, thus there were fewer orthopaedic surgeons included compared to physiotherapists. In total, 101 cases were eligible, where all three parties (patient, orthopaedic surgeon and physiotherapist) had answered their specific questionnaire about the decision-making process.

There were no differences between the 101 patients who were included in the current study and the remaining 174 patients from the NACOX study regarding age, sex or Tegner Activity Scale score before injury. However, there was a significantly (*p* = 0.03) higher proportion of ACLR among the included patients (63% in the whole NACOX cohort and 71% in the present study cohort).

Among the 101 included patients, a non-surgical treatment decision was taken for 29%, and an ACLR treatment decision for 71%. Five patients had a previous ACL injury on the contralateral knee. ACLR was performed at a mean of 5.2 months (SD 4.9, range 1–12) after injury. Among the patients where an ACLR treatment decision was taken, 7 patients reported that a new injury occurred before the treatment decision, that could have contributed to the ACLR decision. None of the new injuries required surgery, but where mainly giving ways. For 3 patients a decision for ACLR was taken, but they did not undergo ACLR. The reasons for this varied: one patient believed the knee function was good enough to decline surgery when it was offered, one had an incomplete ACL tear and for the third patient, the reason for not undergoing surgery was unknown. There were 4 patients where a non-ACL decision was taken, that stated that they would have chosen ACLR if they were to choose again, asked at 12 months after the injury. None of these 4 patients had a new injury before the treatment decision was taken, and none of them disagreed about the non-ACLR decision at the time of the treatment decision. Among the 7 patients that stated that they would not have chosen ACLR if they were to choose again at 12 months after surgery, none of them had a new injury nor stated that they disagreed with their treatment decision at the time it was taken.

The IKDC-SKF mean scores did not differ between the non-ACLR group (78, SD15) at 12 months after injury, and the ACLR group (77, SD17) at 12 months after ACLR. At the time of the treatment decision, the vast majority of patients reported that the chosen treatment matched their preferences (89% for the non-ACLR group and 96% for the ACLR group). At 12 months after injury or ACLR, most of the patients reported that they would choose the same treatment again (82% non-ACLR and 84% ACLR) (Table 1).

**Table 1:** Demographic and outcome data (numbers and valid percentages)

	**Total cohort (***n* = 101)	**Non-surgically treated ACL injury ***n* = 29	**ACLR treatment ***n* = 72
Sex; Men (%)/Women (%)	45 (45%)/56 (55%)	17 (59%)/12 (41%)	28 (39%)/44 (61%)
Age at injury (years); mean (SD)	24 (6)	29 (7)	24 (6)
Time from injury to treatment decision (days); mean (SD)	135 (61)	143 (60)	132 (63)
Tegner Activity Score Scale before injury; median (IQR)	7 (7)	4 (7)	8 (7)
IKCD-SKF score 12 months after injury or ACL reconstruction; mean (SD)		78 (15) *n* = 23	77 (17) *n* = 50
Did the treatment decision match the patients’ preferences?^a^	Yes, *n* = 85 (94%)	Yes, *n* = 16 (89%)	Yes, *n* = 69 (96%)
Yes/No (%)^b^	No, *n* = 5 (6%)	No, *n* = 2 (11%)	No, *n* = 3 (4%)
	*n* = 90	*n* = 18	*n* = 72
Would the patient choose the same treatment again?^c^		Yes, *n* = 18 (82%)	Yes, *n* = 39 (84%)
Yes/No (%)^b^		No, *n* = 4 (18%)	No, *n* = 7 (15%)
		*n* = 22	*n* = 46

^a^ reported at the time when treatment decision was made

^b^ percentage of valid yes/no responses

^c^ asked at 12 months after injury or ACLR

For 2 out of every 3 non-ACLR patients (67%) and 4 out of every 5 ACLR patients (79%) were “to a high extent” satisfied with the *information* they got from the orthopaedic surgeon, while the vast majority were satisfied with the information from the physiotherapist (96% and 97%, respectively, reported “to a high extent”). The majority of both the orthopaedic surgeons (97% and 96%) and the physiotherapists (88% and 90%) rated “to a high extent”, that they believed that the patient had understood the information that was given (Table 2).

**Table 2:** Patients’, orthopaedic surgeons’ and physiotherapists’ views on the decision-making process. Response alternatives are: “to a high extent” (includes “to a very high extent” and “to a high extent”), “neither high nor low”, and “to a low extent” (includes “to a low extent”, and “to a very low extent”). The responder to the question is marked in bold letters

	**Non-ACLR ***n* = 29				**ACLR ***n* = 72			
	**To a high extent** valid n (%)	**Neither high nor low extent** valid n (%)	**To a low extent** valid n (%)	**n **total valid responses ^a^	**To a high extent** valid n (%)	**Neither high nor low extent **valid n (%)	**To a low extent **valid n (%)	**n **total valid responses ^a^
**Information**								
To what extent was the **patient** satisfied with the information received during the doctor’s appointment?	18 (67)	7 (26)	2 (7)	27	55 (79)	12 (17)	3 (4)	70
To what extent did the **orthopaedic surgeon** think the patient understood/took in the information given about the choice of treatment?	27 (97)	1 (3)	0	29	63 (96)	2 (3)	1 (1)	66
To what extent was the **patient** satisfied with the information received about the knee injury from the physiotherapist?	26 (96)	1 (4)	0	27	65 (97)	2 (3)	0	67
To what extent did the **physiotherapist** think the patient understood/took in the information given about the choice of treatment?	22 (88)	3 (12)	0	25	60 (90)	6 (9)	1 (2)	67
**Being heard**								
To what extent did the **patient** feel he/she was given the opportunity to explain what was important in the meeting with the orthopaedic surgeon?	18 (75)	3 (13)	3 (13)	24	61 (90)	5 (7)	2 (3)	68
To what extent did the **patient** feel the orthopaedic surgeon understood what was important to him/her?	21 (78)	3 (11)	3 (11)	27	66 (94)	1 (1)	3 (4)	70
To what extent did the **orthopaedic surgeon** think he/she took into consideration what was important for the patient when deciding on the treatment?	28 (100)	0	0	28	68 (99)	1 (1)	0	69
To what extent did the **patient** feel he/she was able to let the physiotherapist know what was important to him/her?	25 (93)	1 (4)	1 (4)	27	58 (97)	2 (3)	0	60
To what extent did the **patient** feel the physiotherapist understood what was important to him/her?	27 (100)	0	0	27	59 (98)	1 (2)	0	60
To what extent did the **physiotherapist** think he/she took into consideration what was important for the patient when deciding on their treatment?	19 (79)	5 (21)	0	24	60 (91)	6 (9)	0	66
**Involvement**								
To what extent did the **patient** feel involved in the decision about treatment?	16 (67)	5 (21)	3 (13)	24	68 (97)	1 (1)	1 (1)	70
To what extent did the **orthopaedic surgeon** think the patient felt involved in the decision about treatment?	26 (100)	0	0	26	68 (99)	1 (1)	0	69
To what extent did the **physiotherapist** think the patient felt involved in the decision about treatment?	20 (77)	5 (19)	1 (4)	26	61 (94)	4 (6)	0	65
**Agreement**								
To what extent did the **patient** and the orthopaedic surgeon agree on the decision made about treatment?	18 (78)	4 (17)	1 (4)	23	58 (87)	7 (10)	2 (3)	67
To what extent were the **orthopaedic surgeon** and the patient in agreement about the treatment that was decided?	27 (93)	2 (7)	0	29	69 (97)	1 (1)	1 (1)	71
To what extent did the **patient** and the physiotherapist agree on the decision made about treatment?	17 (81)	4 (19)	0	21	48 (87)	7 (13)	0	55
To what extent were the **physiotherapist** and the patient in agreement about the treatment that was decided?	16 (62)	10 (38)	0	26	47 (73)	13 (20)	4 (6)	64
To what extent were the **orthopaedic surgeon** and the assigned physiotherapist in agreement about the treatment that was decided?	12 (80)	3 (20)	0	15	38 (80)	9 (19)	0	47
To what extent were the **physiotherapist** and the assigned orthopaedic surgeon in agreement about the treatment that was decided?	11 (58)	8 (42)	0	19	31 (66)	11 (23)	5 (10)	47

^a^Responses were given on a Likert scale from “to a low extent” to “to a very high extent”. The rates for other responses (e.g. “I do not know” or “physiotherapist/orthopaedic surgeon was not included in the decision”) are accounted for in Appendix 2

On the topic of *being heard*, 75% of non-ACLR and 90% of ACLR patients rated that they had the *opportunity to explain* to the orthopaedic surgeon what was important to them “to a high extent”. Corresponding numbers for the patients having the opportunity to explain to the physiotherapists were 93% and 97%, respectively. The patients’ rating of whether the orthopaedic surgeons had *understood what was important* to them showed that 78% and 94%, respectively, rated it as “to a high extent”, with the corresponding numbers for physiotherapists being 100% and 98%, respectively. All orthopaedic surgeons (100% and 99%) rated that *they considered the patient’s wishes* regarding the choice of treatment “to a high extent”, with corresponding numbers for physiotherapists being 79% and 91%, respectively (Table 2).

Most patients felt *involved* in the treatment decision process, although fewer patients with a decision for non-ACLR (67%) rated being involved “to a high extent”, compared to the ACLR group (97%). Most orthopaedic surgeons rated “to a high extent” the patients’ involvement (100 and 99%, respectively), while slightly fewer of the physiotherapists rated the patients’ being involved “to a high extent” for the non-ACLR group (77% and 94%, respectively) (Table 2).

With regards to the *agreement* between the three parties in the treatment decision (patient, orthopaedic surgeon, physiotherapist), fewer patients in the non-ACLR group reported agreement “to a high extent” (78% reported high agreement with the orthopaedic surgeon and 81% with the physiotherapist), compared to patients in the ACLR group (87% reported high agreement with the orthopaedic surgeon and 87% with the physiotherapist). Fewer physiotherapists (58% in the non-ACLR group and 66% in the ACLR group) reported agreement with the orthopaedic surgeon “to a high extent”, compared to the orthopaedic surgeons’ (80% in both non-ACLR and ACLR group) reports about agreement with physiotherapists.

## Discussion and conclusion

### Discussion

The present study, according to the authors’ knowledge, is unique of its kind. It aims to investigate and present three perspectives on the treatment decision-making process after an ACL injury, those of patients and their orthopaedic surgeons and physiotherapists. The results show that, in general, patients and caregivers seem to consider that patients’ needs to be informed, heard and involved and to agree on the decision during the treatment process are fulfilled to a high extent. Fewer patients in the non-ACLR decision group gave a high rating for their contact with the orthopaedic surgeon compared to the ACLR group. Orthopaedic surgeons rated generally highly in all categories.

In total, more than two thirds of orthopaedic surgeons and physiotherapists gave high ratings for the questions about *information* and about *being heard.* Most patients in the ACLR group gave high ratings to these questions; however, about one patient in every three in the non-ACLR group, compared to one patient in every five in the ACLR group, were less content with the information. The same tendency was found in the questions about patients’ *involvement*, where fewer patients in the non-ACLR decision group (67%) gave high ratings compared to the ACLR group (97%). These results indicate that, when a treatment decision-making process results in ACLR as treatment, more patients perceive that they have *been heard* and *involved* than when a non-ACLR treatment decision is taken.

Concerning decisions on the choice of treatment, 78% of the non-ACLR group and 87% of the ACLR group agreed with the orthopaedic surgeon about the treatment. This result is somewhat surprising, since all treatments require agreement between all the parties involved before they can commence. Overall, more orthopaedic surgeons rated these questions more highly than the patients.

In terms of the caregivers’ rating of patient involvement, 100% of orthopaedic surgeons thought patients were involved “to a very high extent”. It has previously been suggested that a non-surgical treatment decision taken by an orthopaedic surgeon is related to greater experience and a less macho attitude towards surgery [27], yet the present results show that patients and orthopaedic surgeons seem to have different opinions about the decision-making process, in terms of patient involvement. Orthopaedic surgeons are in general willing to be involved in a shared decision-making process [28], although it is a barrier that they are concerned that it is more time consuming [29]. Research shows that both healthcare personnel and patients prefer a SDM management [30], and when they are provided with information about the injury and treatment options, patients are more likely to be involved in their healthcare decisions [31]. A treatment decision-making process where SDM and patient involvement is practised is extremely important in order to strive for and improve patient-centred care [32]. This, in light of the results from the present study, emphasises the importance of putting extra effort into the non-ACLR treatment decision-making process, to enhance patient involvement.

The majority of patients in the ACLR group stated that the treatment decision matched their preferences, but there were more missing data in the non-ACLR group (*n* = 11, 37%), and therefore uncertainty as to whether the non-ACLR decision was the preferred treatment. One year after ACLR, seven patients reported that they would not choose ACLR again if they had to make a new treatment choice. A surgical treatment cannot be undone and that can be a reason for giving the patient some time to consider the treatment options and allow for an appropriate share of decision-making. The IKDC-SKF scores show that the patients seem to have acceptable symptoms, function and sports activity level [25] at twelve months after injury for non-ACLR and twelve months after surgery for ACLR, which indicates that the results of the treatments were successful.

Compared to physiotherapists’ ratings, more of the orthopaedic surgeons rated positively that patients understood the *information* they had been given and that they took the patient’s wishes into consideration, as well as that the patient felt *involved* in the decision. On the other hand, a greater proportion of patients gave high ratings to the *information* given, and the questions about *being heard* in the meeting with their physiotherapist, compared to the meeting with their orthopaedic surgeon. This indicates that there is a discrepancy between how patients and caregivers experience these meeting(s) and the process of treatment decision-making. A potential difference in patients’ experience is that a structured rehabilitation might include several meetings between the patient and physiotherapist, while an orthopaedic surgeon only meets the patient once or a few times. Lack of time and concern about interference with workflow are factors that have been shown to be barriers for practising SDM in orthopaedic clinics, although a meeting where the conversation is based on an SDM approach might not be more time consuming [29].

Orthopaedic surgeons and physiotherapists gave high ratings to the statement that they agreed about the choice of treatment, but this was done by fewer physiotherapists (58–66%) than orthopaedic surgeons (80%). However, there was a high number of responses stating that the physiotherapist or orthopaedic surgeon was not involved in the decision. Previous research has shown that the orthopaedic surgeon might not always be involved in a non-ACLR decision [8], and perhaps a physiotherapist is not always involved when an early decision for ACLR is taken. In contrast, orthopaedic surgeons and physiotherapists do state that they rate the importance of each other’s assessments highly [6]. Since it is proposed that a structured rehabilitation should be initiated in most situations before the treatment decision is made [5], it is likely that the physiotherapist will have had repeated contact with the patient before the treatment decision takes place. The physiotherapist will therefore have had the opportunity to discuss the preferred treatment and expectations with the patient. Our results show that there might be room for improvement in the interprofessional communication between physiotherapists and orthopaedic surgeons. Panesar et al. [33] found that the causes of adverse events in an orthopaedic setting are often related to poor teamwork and poor communication, which emphasises the importance of interprofessional communication.

The choice of surgical treatment after an ACL injury is an elective surgery with a possible quality-of-life enhancing result, rather than a treatment for a life-threatening situation or condition. In such situations, many factors might affect patients’ preferences and expectations, depending on the state of the diagnosis and patient characteristics [34]. Discussions of preoperative expectations, as well as postoperative reality, is suggested to be an important part of clinical care [35]. The results of the present study show that the majority of patients with an ACL injury were satisfied with the information they received from both their orthopaedic surgeon and their physiotherapist. Most patients also experienced that they were given the opportunity to explain what was important to them, and that the healthcare professionals listened. However, fewer patients with a non-ACLR treatment decision seem to have felt that they were involved in the decision to choose treatment. This calls for action to understand what patients need during the decision-making process in order to experience involvement and how healthcare personnel can enhance patient involvement.

There are some limitations to this study. Since healthcare systems might be structured differently in different parts of the world, the results may not be applicable everywhere, but they are probably valid in Scandinavian settings. However, there should be efforts made to enhance an SDM process during treatment decisions in every healthcare encounter, thus the discussion should be brought into the light regardless of the healthcare structure.

The SDMP questionnaire developed for this study was based on litteratur on SDM och inspired by the collaboRATE framework. To address the specific treatment decision situation, and all parties involved, no existing questionnaire was adequate to authors knowledge, but new questions needed to be developed. The SDMP was tested for face validity by healthcare professionals. More test of validity and reliability could have provided more explicit indications of the fit of the questionnaire, in the specific population and to the research questions. One of the questions in the SDMP differ slightly between the health care personnel questionnaire and the patient questionnaire. It was adapted to get more accurate information from each party, it concerns. The question about whether the healthcare personnel took patient’s wishes into consideration in their decision on treatment, was in the patient questionnaire represented by two questions: if they were able to let the personnel know what was important to them and if the personnel understood what was important to them. A direct mirroring of the questions to the patient (i.e., asking the healthcare personnel if they allowed the patient to explain what was important and if they listened to the patient) was considered not to give as much information about the interaction between the three parties; therefore, the question about taking patient’s wishes into consideration was chosen.

The patients in the present study had all suffered an ACL injury, but the inclusion criteria also allowed for associated injuries, as well as a previous ACL injury to the other knee. This allows for greater heterogeneity in the study population, with greater variation in the patients’ preunderstanding of the situation that they bring into the meeting. However, it also reflects the clinical reality, as associated injuries and previous ACL injuries to the other knee are common among ACL injured patients. The analyses show no differences in age, sex and activity levels before injury between the selected group of patients in the NACOX study who fulfilled the inclusion criteria for the current analyses compared to the patients who were excluded. This indicates that the participants in the present study can be considered representative regarding these aspects. The slightly higher proportion of ACLR in the selected group for the current analyses may be due to the fact that sometimes one or more of the parties, especially the orthopaedic surgeon, are not part of a non-surgical treatment.

This study is an explorative descriptive study, and the results are presented as differences in fixed numbers, not statistical differences. A larger study population could provide the opportunity to conduct other statistical analyses and examine statistical differences to a greater extent.

The quantitative approach that was used allows quantification of the data and the possibility to draw conclusions about the population with ACL injuries. A qualitative approach, however, would have given a deeper understanding of how patients, orthopaedic surgeons and physiotherapists experience the treatment decision-making process, and could form a future complement to the results gathered in the present study.

## Conclusion

Regardless of treatment, most patients and caregivers consider that patients’ needs to be *informed*, *heard* and *involved* and *to agree* of the decision about the treatment process are fulfilled to a great extent. However, patients for whom a non-ACLR decision is made experience being involved in the treatment decision to a lower extent.

### Practice implications

The non-ACLR treatment decision process should be clarified further, especially from the patient involvement perspective.

## Supplementary Information

**Additional file 1.**

**Additional file 2.**

## Data Availability

All data and materials are available and can be retrieved via contact with corresponding author**.**
