# Supplementary material for: Treatment decision-making process after an anterior cruciate ligament injury: patients’, orthopaedic surgeons’ and physiotherapists’ perspectives
Source: BMC Musculoskelet Disord. 2022 Aug 16;23:782. doi: 10.1186/s12891-022-05745-4 (PMC9380364; doi:10.1186/s12891-022-05745-4)
Supplement: Supplementary file 1 — Additional file 1. [file 12891_2022_5745_MOESM1_ESM.docx]

**Shared Decision Making Process Questionnaire – patient**

**1. Is the treatment chosen in accordance with your wishes?**

- Yes
- No

The questions below are about the *decision-making process* for choosing how your knee injury should be treated

**To what extent were you satisfied with the information you received during your doctor’s appointment?**

- To a very high extent
- To a high extent
- Neither high nor low extent
- To a low extent
- To a very low extent
- Have not had a doctor’s appointment
- Don’t know

**To what extent were you satisfied with the information you received about your knee injury from the physiotherapist?**

- To a very high extent
- To a high extent
- Neither high nor low extent
- To a low extent
- To a very low extent
- Have not seen a physiotherapist
- Don’t know

**To what extent do you feel that you were given the opportunity to present what was important to you in the meeting with the orthopaedic surgeon?**

- To a very high extent
- To a high extent
- Neither high nor low extent
- To a low extent
- To a very low extent
- Don’t know
- I was not able to communicate what was important to me
- No orthopaedic surgeon involved in the decision
- Don’t know

**To what extent do you feel the orthopaedic surgeon understood what was important to you?**

- To a very high extent
- To a high extent
- Neither high nor low extent
- To a low extent
- To a very low extent
- Don’t know

**To what extent do you feel you were given the opportunity to present what was important to you in the meeting with the physiotherapist?**

- To a very high extent
- To a high extent
- Neither high nor low extent
- To a low extent
- To a very low extent
- I was not able to communicate what was important to me
- No physiotherapist involved
- Don’t know

**To what extent did you feel the physiotherapist understood what was important to you?**

- To a very high extent
- To a high extent
- Neither high nor low extent
- To a low extent
- To a very low extent
- Don’t know

**To what extent did you feel involved in the decision about your treatment?**

- To a very high extent
- To a high extent
- Neither high nor low extent
- To a low extent
- To a very low extent
- Don’t know
- I did not feel involved

**To what extent did you and the orthopaedic surgeon agree on the decision made about treatment?**

- To a very high extent
- To a high extent
- Neither high nor low extent
- To a low extent
- To a very low extent
- We did not agree
- No orthopaedic surgeon involved in the decision
- Don’t know

**To what extent did you and the physiotherapist agree on the decision made about treatment?**

- To a very high extent
- To a high extent
- Neither high nor low extent
- To a low extent
- To a very low extent
- We did not agree
- Don’t know
- No physiotherapist involved in the decision

**Shared Decision Making Process Questionnaire – Orthopaedic surgeon**

Please find below a number of questions relating to the decision-making process in the choice of treatment for the patient with an anterior cruciate ligament injury in question.

**To what extent do you think the patient understood/took in the information you gave them about the choice of treatment?**

- To a very high extent
- To a high extent
- Neither high nor low extent
- To a low extent
- To a very low extent
- Don’t know

**To what extent do you think you took into consideration what was important for the patient when deciding on their treatment?**

- To a very high extent
- To a high extent
- Neither high nor low extent
- To a low extent
- To a very low extent
- Don’t know

**To what extent do you think the patient felt involved in the decision about treatment?**

- To a very high extent
- To a high extent
- Neither high nor low extent
- To a low extent
- To a very low extent
- Don’t know

**To what extent did you and the patient agree about the treatment that was decided?**

- To a very high extent
- To a high extent
- Neither high nor low extent
- To a low extent
- To a very low extent
- Don’t know

**To what extent did you and the assigned physiotherapist agree about the treatment that was decided?**

- To a very high extent
- To a high extent
- Neither high nor low extent
- To a low extent
- To a very low extent
- Don’t know
- No physiotherapist involved

**Shared Decision Making Process Questionnaire – Physiotherapist**

Please find below a number of questions relating to the decision-making process in the choice of treatment for the patient with an anterior cruciate ligament injury in question.

**To what extent do you think the patient understood/took in the information you gave them about the choice of treatment?**

- To a very high extent
- To a high extent
- Neither high nor low extent
- To a low extent
- To a very low extent
- Don’t know

**To what extent do you think you took into consideration what was important for the patient when deciding on their treatment?**

- To a very high extent
- To a high extent
- Neither high nor low extent
- To a low extent
- To a very low extent
- Don’t know

**To what extent do you think the patient felt involved in the decision about treatment?**

- To a very high extent
- To a high extent
- Neither high nor low extent
- To a low extent
- To a very low extent
- Don’t know

**T what extent were you and the patient in agreement about the treatment that was decided?**

- To a very high extent
- To a high extent
- Neither high nor low extent
- To a low extent
- To a very low extent
- Don’t know

**To what extent were you and the assigned orthopaedic surgeon in agreement about the treatment that was decided?**

- To a very high extent
- To a high extent
- Neither high nor low extent
- To a low extent
- To a very low extent
- Don’t know
- No orthopaedic surgeon involved in the decision
