# Supplementary material for: Treatment decision-making process after an anterior cruciate ligament injury: patients’, orthopaedic surgeons’ and physiotherapists’ perspectives
Source: BMC Musculoskelet Disord. 2022 Aug 16;23:782. doi: 10.1186/s12891-022-05745-4 (PMC9380364; doi:10.1186/s12891-022-05745-4)
Supplement: Supplementary file 2 — Additional file 2. [file 12891_2022_5745_MOESM2_ESM.docx]

Appendix 2. All answer options and ratings described for the Shared Decision-Making Process Questionnaire given to patients, orthopaedic surgeons and physiotherapists.

|  |  |  | | | |  | | | |  |  |
| --- | --- | --- | --- | --- | --- | --- | --- | --- | --- | --- | --- |
| **Information** |  | To a very high extent | To a high extent | Neither a high nor low extent | To a low extent | To a very low extent | Don´t know | Other option^a^ | No orthopaedic surgeon involved in the decision | No physiotherapist involved in the decision | N total |
| To what extent was the **patient** satisfied with the information received during the doctor’s appointment? | Non-ACLR  ACLR | 8  32 | 10  23 | 7  12 | 0  1 | 2  2 | 2  1 | -  - | -  - | -  - | 29  71 |
| To what extent did the **orthopaedic surgeon** think the patient understood/took in the information given about the choice of treatment? | Non-ACLR  ACLR | 13  37 | 15  26 | 1  2 | 0  1 | 0  0 | 0  0 | -  - | -  - | -  - | 29  66 |
| To what extent was the **patient** satisfied with the information received about the knee injury from the physiotherapist? | Non-ACLR  ACLR | 17  40 | 9  25 | 1  2 | 0  0 | 0  0 | 2  3 | -  - | -  - | -  - | 29  70 |
| To what extent did the physiotherapist think the patient understood/took in the information given about the choice of treatment? | Non-ACLR  ACLR | 8  34 | 14  26 | 3  6 | 0  1 | 0  0 | 1  4 | -  - | -  - | -  - | 26  71 |
| **To be heard** |  |  |  |  |  |  |  |  |  |  |  |
| To what extent did the **patient** feel he/she was able to let the orthopaedic surgeon know what was important in the meeting? | Non-ACLR  ACLR | 8  51 | 10  28 | 3  8 | 1  2 | 1  1 | 1  3 | 1^b^  2^b^ | 0  1 | -  - | 25  96 |
| To what extent did the **patient** feel the orthopaedic surgeon understood what was important to him/her? | Non-ACLR  ACLR | 12  41 | 9  25 | 3  1 | 0  1 | 3  2 | 2  0 | -  - | -  -^c^ | -  - | 29  70 |
| To what extent did the **orthopaedic surgeon** think he/she took into consideration what was important for the patient when deciding on their treatment? | Non-ACLR  ACLR | 14  48 | 14  20 | 0  1 | 0  0 | 0  0 | 0  0 | -  - | -  - | -  - | 28  69 |
| To what extent did the **patient** feel he/she was able to let the physiotherapist know what was important to him/her? | Non-ACLR  ACLR | 15  45 | 10  13 | 1  2 | 1  0 | 0  0 | 2  1 | 0^b^  0^b^ | -  - | 0  10 | 29  71 |
| To what extent did the **patient** feel the physiotherapist understood what was important to him/her? | Non-ACLR  ACLR | 17  45 | 10  14 | 0  1 | 0  0 | 0  0 | 2  1 | -  - | -  - | -  -^d^ | 29  61 |
| To what extent did the **physiotherapist** think he/she took into consideration what was important for the patient when deciding on their treatment? | Non-ACLR  ACLR | 10  38 | 9  22 | 5  6 | 0  0 | 0  0 | 2  4 | -  - | -  - | -  - | 26  70 |
| **Involvement** |  |  |  |  |  |  |  |  |  |  |  |
| To what extent did the **patient** feel involved in the decision about treatment? | Non-ACLR  ACLR | 9  56 | 7  12 | 5  1 | 0  0 | 2  0 | 1  1 | 1^e^  1^e^ | -  - | -  - | 25  71 |
| To what extent did the **orthopaedic surgeon** think the patient felt involved in the decision about treatment? | Non-ACLR  ACLR | 10  51 | 16  17 | 0  1 | 0  0 | 0  0 | 0  0 | -  - | -  - | -  - | 26  69 |
| To what extent did the **physiotherapis**t think the patient felt involved in the decision about treatment?  **Agreement** | Non-ACLR  ACLR | 6  33 | 14  28 | 5  4 | 1  0 | 0  0 | 1  4 | -  - | -  - | -  - | 27  69 |
| To what extent did the **patient** and the orthopaedic surgeon agree on the decision made about treatment? | Non-ACLR  ACLR | 15  43 | 3  15 | 4  7 | 0  0 | 0  1 | 1  2 | 1^f^  1^f^ | 1  2 | -  - | 25  71 |
| To what extent were the **orthopaedic surgeon** and the patient in agreement about the treatment that was decided? | Non-ACLR  ACLR | 16  49 | 11  20 | 2  1 | 0  1 | 0  0 | 0  0 | -  - | -  - | -  - | 29  71 |
| To what extent did the **patient** and the physiotherapist agree on the decision made about treatment? | Non-ACLR  ACLR | 13  29 | 4  19 | 4  7 | 0  0 | 0  0 | 0  2 | 0^f^  0^f^ | -  - | 3  14 | 24  71 |
| To what extent were the **physiotherapist** and the patient in agreement about the treatment that was decided? | Non-ACLR  ACLR | 9  29 | 7  18 | 10  13 | 0  2 | 0  2 | 1  6 | 0^f^  0^f^ | -  - | -  - | 27  70 |
| To what extent were the **orthopaedic surgeon** and the assigned physiotherapist in agreement about the treatment that was decided? | Non-ACLR  ACLR | 3  21 | 9  17 | 3  9 | 0  0 | 0  0 | 4  17 | - | - | 10  5 | 29  69 |
| To what extent were the **physiotherapist** and the assigned orthopaedic surgeon in agreement about the treatment that was decided? | Non-ACLR  ACLR | 6  20 | 5  11 | 8  11 | 0  1 | 0  4 | 4  22 | - | 2  2 | -  - | 25  71 |

^a^ The response option was slightly different depending on the question, and is described for each question where applicable.

^b^ “I was not able to communicate what was important to me.”

^c^ The question was not asked to the one respondent who answered that an orthopaedic surgeon was not involved, in the previous question.

^d^ The question was not asked to the 10 respondents who answered that a physiotherapist was not involved, in the previous question.

^e^ “I did not feel involved.”

^f^ “We did not agree.”
